# Fungal contamination of medical students’ mobile phones from the University of Belgrade, Serbia: a cross-sectional study

**DOI:** 10.1038/s41598-022-21118-2

**Published:** 2022-10-07

**Authors:** Eleonora Dubljanin, Teodora Crvenkov, Isidora Vujčić, Sandra Šipetić Grujičić, Jakša Dubljanin, Aleksandar Džamić

**Affiliations:** 1 Faculty of Medicine, Institute of Microbiology and Immunology, University of Belgrade, Belgrade, Serbia; 2 Faculty of Medicine, University of Belgrade, Belgrade, Serbia; 3 Faculty of Medicine, Institute of Epidemiology, University of Belgrade, Belgrade, Serbia; 4 Department of Cardiology, University Clinical Hospital Center Zemun, Belgrade, Serbia

**Keywords:** Fungi, Disease prevention

## Abstract

The study aimed to characterize fungal contamination of medical students’ mobile phones, investigate mobile phones’ usage and cleaning habits, identify independent risk factors for fungal contamination, and awareness of mobile phones as a potential route of infection. In a cross-sectional study, medical students’ mobile phones were sampled for possible fungal contamination. The questionnaire was used to record mobile phone usage, cleaning habits, and awareness of mobile phones as a source of infection. A total of 492 medical students were included and fungal contamination of mobile phones was confirmed in 32.11%. The most frequent fungal isolates on students’ mobile phones were *Candida albicans* (28.5%), followed by *Aspergillus niger* (11.4%), and *Penicillium chrysogenum* (9.5%). Factors independently associated with fungal contamination of students’ mobile phones were: lack of mobile phone cleaning (OR = 0.381; *p* < 0.001), and usage of mobile phones near patients’ beds (OR = 0.571; *p* = 0.007). The results of this study confirmed that students who use their mobile phones in hospital wards have a higher rate of fungal contamination. The development of active surveillance and preventive strategies is needed to reduce the risk of cross-contamination and increase awareness of fungal transmission via mobile phones.

## Introduction

During the last decade, mobile phones (both keypads and smartphone devices) become one of the most common items people carry around in both professional and personal settings^1^. The usage of mobile phones has spread abruptly throughout the world and dramatically influenced the way of communication, access to information, and lifestyle in general^2^. The number of mobile users in 2021 is estimated to be 7.1 billion with forecasts suggesting this is likely to rise to 7.49 billion by 2025^3^. It is estimated that 90% of teenagers in Europe and Asia^4^, and 86–94% of individuals of both genders aged below 65 years, have their mobile phones^5^.

Nowadays, mobile phones have evolved from just a talking medium to a multipurpose device and become one of the most essential accessories, being affordable and easy to handle^6^. The medical profession has integrated mobile phones as an important means of communication among healthcare workers^7^. Besides enhancing clinician communication, mobile phones provide instant access to unlimited resources and have the potential to transform traditional uses of imaging, sensing, and diagnostic systems, especially for point-of-care applications and field settings^8^.

However, mobile phones come into close contact with their users and carry the personal microbiome of their owners^9^. Mobile phones are now used almost everywhere resulting in continuous exposure to different types of microorganisms^10^. Moreover, moving constantly with its owner mobile phones of health care professionals can be easily contaminated with microorganisms from the hospital environment^6^. Mobile phones can be reservoirs for many microorganisms and a potential vehicle for the spread of pathogenic microorganisms in hospital settings^11^. Constant handling, the heat generated from mobile phones, and the cervices of cracked screens and phone covers provide excellent habitats for colonization with different microorganisms^2^.

There is growing evidence that mobile phones in clinical settings are highly contaminated with different microorganisms and could, like other contaminated fomites and surfaces, play a key role in the spread of nosocomial infections^12^. Different kinds of microorganisms can be isolated from the surface of mobile phones that can be part of normal skin microbiota, but the isolation of pathogens causing nosocomial infections has also been frequently reported such as *Staphylococcus aureus*, coagulase-negative staphylococci, *Escherichia coli, Candida* spp., etc.^12^. Microorganisms that can be transmitted from mobile phones represent a potential risk, especially for patients in intensive care units, who are at very high risk of nosocomial infections due to a high number of invasive devices (e.g., intravascular catheters) that can facilitate the entry of pathogens^12^.

Many studies investigate the contamination of mobile phones by bacteria^1,4,6,10–12^, but contamination of mobile phones by fungi has been poorly investigated. In the present study, we hypothesized that students who use their mobile phones in hospital wards have a higher rate of fungal contamination.

This study aimed to characterize the fungal contamination of medical students’ mobile phones. First, we examined which fungi are most frequently isolated from students’ mobile phones. Second, we investigate the details of mobile phone usage, and identified independent risk factors for fungal contamination. Third, we analyzed the extent and mode of mobile phone cleaning habits. Finally, we surveyed students’ awareness of mobile phones as reservoirs as well as potential routes for infection.

## Materials and methods

### Study design and participants

A cross-sectional study was conducted on medical students at the Faculty of the Medicine University of Belgrade from 15th October to 20th December 2019. The study population included 4^th^-grade students practicing mostly clinical subjects in different clinical wards recruited by the convenience sampling method. Data from students, as well as samples for laboratory diagnosis, were collected in lab rooms at the Institute of Microbiology and Immunology through an anonymous questionnaire administered before the start of compulsory practical lessons in Clinical Microbiology. The required sample size was calculated to be 143, with a test power of 0.8, and a two-sided α level of 0.05.

All the participants were informed about the goals of the research and informed consent was obtained from each participant included in the study. We excluded students who refuse to get their mobile phones sampled for fungal contamination and students who returned the incompletely filled questionnaire. The study was approved by the Ethics Committee of the Faculty of Medicine University of Belgrade (1322/VII-45). All methods were performed following the relevant guidelines and regulations.

An anonymous questionnaire was used to record the cleaning habits and mobile phone usage details of participants. The questionnaire was designed for the purpose of the study by two researchers (ED, TC). To ease understanding and interpretation of the items, a pilot study was conducted among 20 medical students at the University of Belgrade, and their suggestions were incorporated into the final version. The questionnaire included basic demographic data (age, gender, place of living), academic data (study year), mobile phone usage data (duration of possession of the mobile phone, frequency of replacement, frequency of daily usage of mobile phone, place of holding a mobile phone in home/faculty/clinic), as well as mobile phone cleaning habits (frequency and way of cleaning).

Regarding the frequency of daily mobile phone usage students could choose a couple of times a day – mainly basic use of answering/calling and texting, periodically – if there is time during a day at least a couple of hours without using a device, and constantly – continuously usage of mobile phone for different purposes (web searching, social media, gaming, mailing…).

Regarding the frequency of cleaning students could choose daily – cleaning at least once daily, weekly – cleaning at least once weekly, and occasionally – irregular cleaning mostly where there is evident filth on a mobile phone.

The questionnaire also included questions regarding awareness that mobile phones can serve as a source of infection. Students were asked to declare if they regard mobile phones as a potential reservoir of infection, practice hand hygiene after handling a mobile phone, and have ever superficial fungal infection in a lifetime.

### Sampling and laboratory procedures

Every student included in the study consented to have their mobile phone sampled for possible fungal contamination. A sterile cotton swab immersed in sterile saline was rolled over the front, back and lateral side of the mobile phone including buttons of keypads, mouth and earpiece, volume and lock keys, audio, and battery charger jack, to cover the entire surface of the device. Phones that were in personalized cases were not removed from the casing and the external surface was swabbed. All the surfaces were thoroughly swabbed and swabs were immediately inoculated on Sabouraud dextrose agar (HiMedia, Mumbai, India). Inoculated media were transported to Medical Mycology Laboratory at the Institute of Microbiology and Immunology, Faculty of the Medicine University of Belgrade where all the laboratory testing was done.

Inoculated plates were incubated for 21 days at 30 ± 2 °C, and checked weekly for fungal growth. If fungal growth was observed, the causative agent was identified by colony morphology and microscopic characteristics^13^. Plates with no fungal growth even after 4 weeks of incubation were considered negative.

The identification of dermatophytes and non-dermatophyte moulds was performed by macroscopic and microscopic morphological examination of colonies, and by biochemical methods (urease test) or microculture technique application if required. Yeast isolates were identified by growth on CHROM agar (HiMedia, Mumbai, India) and with the use of the API 20C AUX Commercial System (BioMerieux, Marcy-L’Etoile, France).

### Statistical analysis

A descriptive analysis was used to detail the main characteristics of the study population. Statistical analyses were performed using chi-square and Fisher’s test to compare categorical variables. Mobile phone usage was analyzed by univariate and multivariate logistic regression analysis. In the multivariate analysis, all the variables that correlated with fungal contamination of students’ mobile phones at a significance level of ≤ 0.10 were included. The comparison of means was performed by independent samples *t*-test. The statistical software used was SPSS v15 (SPSS Inc., Chicago, Illinois, USA). *P*-value ˂0.05 was considered to be statistically significant.

## Results

### Demographic description

The study included 492 out of 516 (response rate 95.35%), medical students, in the 4th academic year. The average age of students was 22.63 ± 1.06 (age range 21–29). The majority of students were females 319/492 (64.84%), while 173/492 (35.16%) were male students. Fungal contamination of students’ mobile phones was found in 158/492 (32.11%), while 334/492 (67.89%) were negative for fungal contamination.

**Figure 1:**
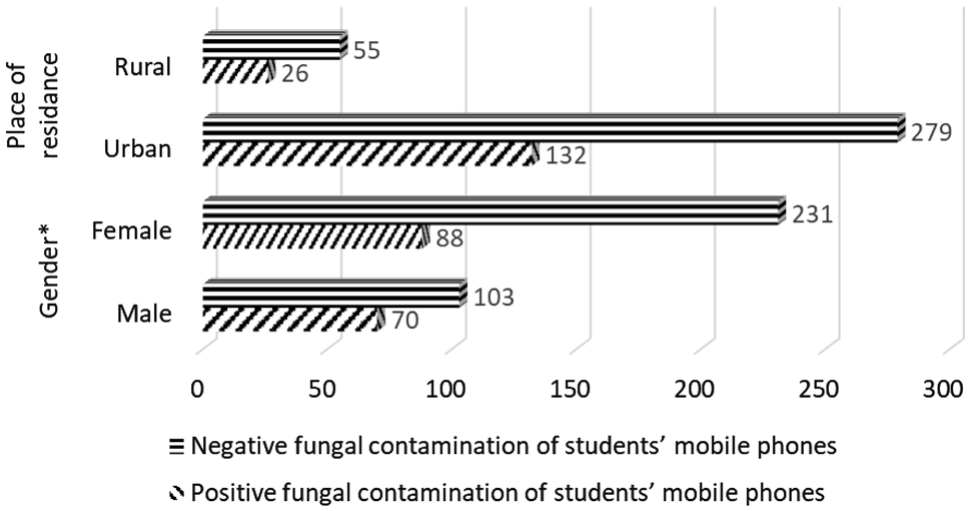
Distribution of demographic characteristics in 492 medical students by presence of fungal contamination of students’ mobile phones; ∗the significant difference between two observed groups.

Male students (*p* = 0.005) had significantly more often mobile phones contaminated with fungi. There was no difference in fungal contamination of students’ mobile phones concerning the place of residence (Fig. 1).

### Mobile phones usage description

The average duration of mobile phone possession in the medical student population was 19.96 ± 16.22 months (duration of possession range from 1–144 months), while the average frequency of mobile phone replacement was 32.84 ± 12.73 months (frequency of replacement range from 12 to 120 months). The majority of medical students had smartphones 488/492 (99.19%), while 4/492 (0.81%) had keypad mobile phones.

Students with fungal contamination of their mobile phones significantly more often use mobile phones near patients’ beds in hospital wards (*p* = 0.007), hold their phones in pockets at university (*p* = 0.038), and keep their mobile phones in random places at home (*p* < 0.001). Also, students with fungal contamination of their mobile phones significantly less often clean their phones compared to students negative for mobile phones’ fungal contamination (*p* < 0.001). There was no difference in fungal contamination of students’ mobile phones concerning the duration of mobile phone possession, the average duration of mobile phone possession, frequency of mobile phone replacement, and frequency of daily mobile phone usage (Table 1).

**Table 1:** Distribution of characteristics regarding mobile phone usage in 492 medical students by presence of fungal contamination on students’ mobile phones.

Characteristics	Positive fungal contamination of students’ mobile phones n = 158	Negative fungal contamination of students’ mobile phones n = 334	*p*-value*
**Duration of mobile phone possession**			0.431
0–6 months	32 (20.2)	83 (24.9)	
6–12 months	40 (25.3)	72 (21.6)	
12–24 months	51 (32.3)	118 (35.3)	
> 24 months	35 (22.2)	61 (18.3)	
**The average duration of mobile phone possession, months**	19.72 ± 14.44	20.06 ± 16.97	0.836
**Frequency of mobile phone replacement**			0.589
< 1 year	6 (3.8)	20 (6.0)	
1–2 years	54 (34.2)	109 (32.6)	
> 2 years	98 (62.0)	205 (61.4)	
**Frequency of daily mobile phone usage**			0.164
Constantly	94 (59.5)	190 (56.9)	
Periodically	49 (31.0)	125 (37.4)	
A couple of times a day	15 (9.5)	19 (5.7)	
**Mobile phone cleaning**			< 0.001
Yes	89 (56.3)	262 (78.4)	
No	69 (43.7)	72 (21.6)	
**Mobile phone holding place at university**			0.038
Bag	51 (32.3)	148 (44.3)	
Pocket	102 (64.6)	176 (52.7)	
Other	5 (3.2)	10 (3.0)	
**Mobile phone holding place at home**			< 0.001
Table	107 (67.7)	271 (81.1)	
Pocket	18 (11.4)	27 (8.1)	
Random	32 (20.3)	24 (7.2)	
Other	1 (0.6)	12 (3.6)	
**Usage of mobile phone near patients’ bed**			0.007
Yes	75 (47.5)	115 (34.4)	
No	83 (52.5)	219 (65.6)	

*According to the univariate logistic regression analysis for categorical variable, and independent samples t-test for continuous variable. Numerical data are presented as mean ± standard deviation, categorical as frequency No. (%).

In the multivariate logistic regression analysis were included four variables that showed statistical significance by univariate logistic regression analysis: mobile phone cleaning, mobile phone holding place at university, mobile phone holding place at home, and usage of mobile phone near patients’ beds. Factors independently associated with fungal contamination of students’ mobile phones were (Table 2): mobile phone cleaning (OR = 0.381; *p* < 0.001), and usage of mobile phones near patients’ beds (OR = 0.571; *p* = 0.007).

**Table 2:** Factors independently associated with fungal contamination of students’ mobile phones – results of multivariate logistic regression analysis.

Variable	OR	95% CI	*p*-value
Mobile phone cleaning	0.381	0.249 – 0.584	< 0.001
Mobile phone holding place at university	1.196	0.820 – 1.746	0.353
Mobile phone holding place at home	1.215	0.952 – 1.550	0.117
Usage of mobile phone near patients’ bed	0.571	0.381 – 0.857	0.007

*OR* odds ratio; *CI* confidence interval.

### Mobile phone cleaning habits

A total of 351/492 (71.34%) students report that they clean their mobile phones. The majority of students cleaned their mobile phones with alcohol 189/351 (53.85%), followed by cleaning with wet wipes 89/351 (25.34%). Regarding the frequency of mobile phone cleaning 243/351 (69.23%) students clean their mobile phones occasionally.

There was no difference in presence of fungal contamination on students’ mobile phones regarding the way and frequency of mobile phone cleaning (Table 3).

**Table 3:** Distribution of characteristics regarding mobile phone cleaning habits in 351 students who clean their phones by the presence of fungal contamination of students’ mobile phones.

Characteristics	Positive fungal contamination of students’ mobile phones n = 89 No. (%)	Negative fungal contamination of students’ mobile phones n = 262 No. (%)	*p*-value*
**Way of cleaning**			
Alcohol	51 (57.3)	138 (52.7)	0.573
Wet wipes	20 (22.5)	69 (26.3)	
Paper tissues	12 (13.5)	44 (16.8)	
Other	6 (6.7)	11 (4.2)	
**Frequency of cleaning**			
Daily	16 (18.0)	43 (16.4)	0.565
Weekly	14 (15.7)	35 (13.4)	
Occasionally	59 (66.3)	184 (70.2)	

*According to the chi-square or Fisher’s test.

### Awareness of mobile phones as a possible route of infection

The majority of students included in the study 412/492 (83.74%) regard mobile phones as a reservoir of infection, but only 167/492 (33.94%) wash their hands after mobile phone usage in the wards, while 107/492 (21.75%) reported having ever superficial fungal infection in a lifetime.

Students with fungal contamination of their mobile phones significantly less often regard mobile phones as reservoirs of infection (*p* = 0.001). There was no difference in fungal contamination of students’ mobile phones regarding having ever in lifetime superficial fungal infection, and washing hands after phone usage in hospital wards (Table 4).

**Table 4:** Distribution of characteristics associated with awareness of mobile phones as a possible route of infection in 492 medical students by presence of fungal contamination on students’ mobile phones.

Characteristics	Positive fungal contamination of students’ mobile phones n = 158 No. (%)	Negative fungal contamination of students’ mobile phones n = 334 No. (%)	p-value*
**Reservoir of infection**			
Yes	119 (75.3)	293 (87.7)	0.001
No	21 (13.3)	16 (4.8)	
Do not know	18 (11.4)	25 (7.5)	
**Wash hands after usage of phone in the hospital**			
Yes	55 (34.8)	112 (33.5)	0.838
No	103 (65.2)	222 (66.5)	
**Have ever SFI**			
Yes	39 (24.7)	68 (20.4)	0.293
No	119 (75.3)	266 (79.6)	

*SFI* a superficial fungal infection.

*According to the chi-square test.

### Fungi isolated from students’ mobile phones

The most frequent fungal isolates on students’ mobile phones were *Candida albicans* in 45/158 (28.5%), followed by *Aspergillus niger* in 18/158 (11.4%), and *Penicillium chrysogenum* in 15/158 (9.5%). Regarding fungal groups, non-dermatophyte moulds were most frequently isolated from students’ mobile phones in 91/158 (57.6%), followed by yeasts in 60/158 (41.1%), while dermatophytes were isolated from just two mobile phones and identified as *Trichophyton rubrum* (1.3%). Among non-dermatophyte moulds, the most frequent were *Aspergillus* spp. isolated in 34/91 (37.4%), of which *Aspergillus niger* was most frequent in 18/34 (52.9%). The most frequent yeast was *Candida* spp. isolated in 60/65 (92.3%), with *Candida albicans* as the most common in 45/60 (75.0%) (Table 5).

**Table 5:** Isolated fungi from 158 students with fungal contamination of their mobile phones.

Isolated fungi	No. (%)
**Yeasts (n = 65)**	
*Candida albicans*	45 (28.5)
*Candida parapsilosis*	6 (3.8)
*Candida krusei*	5 (3.2)
*Candida glabrata*	4 (2.5)
*Rhodotorula* spp.	3 (1.9)
*Geotrichum* spp.	2 (1.3)
**Non-dermatophyte moulds (n = 91)**	
*Aspergillus niger*	18 (11.4)
*Penicillium chrysogenum*	15 (9.5)
*Cladosporium* spp.	11 (6.9)
*Alternaria alternata*	10 (6.3)
*Aspergillus flavus*	9 (5.7)
*Aspergillus fumigatus*	7 (4.4)
*Mucor* spp.	6 (3.8)
*Fusarium oxysporum*	6 (3.8)
*Bipolaris* spp.	5 (3.2)
*Acremonium* spp.	2 (1.3)
*Chrysosporium* spp.	1 (0.6)
*Curvularia* spp.	1 (0.6)
**Dermatophytes (n = 2)**	
*Trichophyton rubrum*	2 (1.3)
**Total**	**158 (100.0)**

## Discussion

In our study fungal contamination of students’ mobile phones was found in 32.11%, while 67.89% were negative for fungal contamination. In the study by Kurli et al., who investigate cultivable microbial diversity on mobile phones fungi were found in 29%^10^. Similar findings were reported from a study from Japan with contamination of 31.7%^14^, and a Nigerian study that revealed 30.6% contamination of university students’ mobile phones^15^. On the other hand, some studies reported a much higher contamination rate of medical students’ mobile phones by 92%, but it was predominantly due to bacterial contamination^7^.

Based on our results, male students had significantly more often mobile phones contaminated with fungi. Similar findings were reported by a study from India which showed a higher rate of mobile phone contamination in males compared to females^16^. This can be attributed to better hand hygiene awareness and practices among female students compared to males^16^.

In the present study, students with fungal contamination of their mobile phones significantly less often clean their phones compared to students negative for mobile phones’ fungal contamination. According to our results, lack of mobile phone cleaning was independently associated with fungal contamination of students’ mobile phones. Sailo et al., found that mobile phones which were never cleaned had more microorganisms^16^. In a study from Saudi Arabia, 44.9% of subjects never cleaned their phones^17^, while in an Indian study up to 97% of subjects do not routinely clean their mobile phones^7^.

In our study majority of medical students clean their mobile phones with alcohol, and a similar was reported in a study conducted by Perez-Cano where the most common cleaning methods were alcohol-based solutions^18^. This is consistent with an Italian study where 38% of health care workers reported occasional cleaning of their mobile phones with alcoholic solutions after work activity^12^. Smibert et al. found a zero percent contamination rate of departmental computer keyboards suggesting that routine daily mobile phone cleaning may be sufficient to reduce device contamination with multidrug-resistant organisms^19^. This is in agreement with the findings that cleaning of mobile phones with antibacterial solutions can significantly reduce contamination with microorganisms^20^. In the present study, no significant association was found between fungal contamination and the way of mobile devices cleaning.

Our results suggest that usage of mobile phones near patients’ beds in hospital wards represents an independent risk factor for fungal contamination. Furthermore, in our study students with fungal contamination significantly more often hold their phones in their pockets at university. This is in agreement with the findings that 88.4% of subjects kept their mobile phone in their pocket^12^. Zakai et al., found that all medical students used their mobile phones at work^21^. Constant handling of mobile phones near patients’ beds was confirmed by a study where 76% of healthcare workers used mobile phones while attending to patients^7^. Usage of mobile phones while attending to patients has major health implications since microorganisms from patients can be transmitted to the mobile phones of health care workers and increase the risk of infection among them and their family members^7^. Furthermore, 38% of mobile phones changed bacterial colonization for at least one microorganism comparing the swab results at the beginning and the end of the work shift^12^. However, proper handwashing and disinfecting after attending to each patient practice just 30% of healthcare workers^7^, while some studies suggest that 60% do not wash their hands after using their mobile phones^12^.

According to results from the present study students with fungal contamination of their mobile phones significantly more often keep mobile phones in random places at home. Some studies found a greater number of microorganisms on mobile phones that were carried inside toilets^16^. This is in agreement with findings that found a significant correlation between the contamination level and usage of mobile phones in toilets and sharing^17^. Galazzi et al., found no difference in the colonization of mobile phones by the place where the phones were kept but is more important how the mobile phone is handled than where is kept^12^. While mobile phone by themselves provides a warm habitat that is suitable for microorganisms to grow, dark places like pockets and handbags together with long hours spent with mobile phones can contribute to the growth of microorganisms due to constant handling^22^.

In our study students with fungal contamination of their mobile phones significantly less often regard mobile phones as a reservoir of infection. Many microorganisms including viruses have been shown to survive on inanimate surfaces for an extended period^23^. The majority of the microorganisms on mobile phones represent a transient population^10^, but different microorganisms can colonize non-living and inert substrates by forming biofilms. The environment in which the individual lives and works may also influence the microbial diversity of mobile phones^10^. However, there is no evidence of a direct relationship between environmental pathogens on mobile phones and the rate of hospital-acquired infections^24^.

According to our results, the most frequent fungal isolates on students’ mobile phones were *Candida albicans*, followed by *Aspergillus niger.* Similar results were reported by Kurli et al., who isolated 20 genera of fungi of which the most common were *Candida* spp.*, Aspergillus* spp.*, Aureobasidium* spp., and *Cryptococcus* spp.^10^. Contamination of mobile phones by fungi has been reported by several studies^25–27^. In our study majority of fungal species belongs to opportunistic pathogens, but we also found strictly pathogenic dermatophyte *Trichophyton rubrum* leading agent of tinea pedis and onychomycosis globally, and in our region^28^. Several isolated fungi are opportunistic pathogens and can colonize the skin and mucous membranes of humans^29^. *Candida* spp. is typical human skin commensal and part of the normal skin microbiota, but can form biofilms on implanted devices, grow in total parenteral nutrition, and cause nosocomial outbreaks^30^. Of particular concern are *Candida krusei,* and *Candida glabrata* which were isolated from students’ mobile phones in our study, together with *Candida auris* which all can exhibit resistance to standard treatment^31^. Fungal infections caused by *Candida*, and *Aspergillus* species are an increasing problem worldwide, associated with very high mortality rates^31^. The majority of isolated non-dermatophyte moulds are ubiquitous microorganisms, indoor and outdoor molds, that are seldom capable to cause invasive disease predominantly in immunocompromised patients. On the other hand, these fungi can represent a health risk of being able to produce mycotoxins, as well as cause respiratory tract allergies^32^.

In the present study contamination of 4th-year students’ mobile phones was significantly more often with non-dermatophyte moulds in comparison to 2^nd^-year students’ mobile phones (Supplementary Table 1). Since the number of 2nd-year students was too small, we cannot generalize these results.

Appropriate measures to improve hygiene practices should include education of medical students to increase awareness of nosocomial infection and its mode of transmission, increasing access to hand hygiene infrastructure and supplies in clinical wards, education on proper usage of disinfection and adequate hand hygiene practice, limiting the usage of mobile phones in hospital settings and introduction of standard protocols for decontamination^33–35^.

The present study had some limitations. First, being a cross-sectional study, the study did not address the effect of period variations. However, the completion of this study in a short period and unannounced microbial investigation of the mobile phones did not affect the change in usage and cleaning habits of mobile phones by participants. Second, this was a single-center study, therefore the random error may have affected our analysis which might limit the generalizability of the findings. Third, the study was not set out to report other sources of microbial contamination. Mobile phones especially in hospital environments could serve as a vehicle for the transmission of many pathogenic organisms including multidrug-resistant bacteria and viruses, which could be of particular interest during the present COVID-19 pandemic. However, fungi are present all around us and can be the cause of various diseases, even life-threatening. These diseases are of particular importance in the population of vulnerable patients who have different comorbidities or risk factors for invasive fungal diseases and who often require hospital treatment. Fourth, isolated fungi were not subjected to antifungal susceptibility testing so the study cannot give insight in presence of resistant and multi-drug resistant fungi. Antifungal resistance is becoming a much more frequent problem that can limit treatment options, particularly in patients at high risk for invasive fungal diseases. However, current laboratory standards for antifungal susceptibility testing are laborious, often lack cut-off values, and require the application of molecular methods for final confirmation of resistance. Finally, we included in the study just fourth-year medical students. In our faculty also 5th and 6th-year students mainly have practice in clinical wards, but they are divided into small groups of three up to five students so it would be much more difficult to reach them. Furthermore, many of them have practice in clinical-hospital centers that are away from Medical Faculty. Practicing in clinical wards may contribute to higher contamination of students’ mobile phones, especially with nosocomial pathogens that can be influenced by the type of clinical ward (surgical, internal medicine, etc.). On the other hand, we did not include a representative sample of students in the preclinical years (first-third) to see whether the difference in fungal contamination is associated with hospital settings.

## Conclusion

Our results highlight that the mobile phones of medical students can be contaminated by a wide range of fungi including common agents of fungal infection in humans. Independent risk factors in our study population were: lack of mobile phone cleaning and usage of mobile phones near patients’ beds. The results of this study confirmed that students who use their mobile phones in hospital wards have a higher rate of fungal contamination. The practical implication of the study results can be reflected in the need for the development of active surveillance and the adoption of efficient and suitable decontamination procedures to lessen the chances of cross-contamination via mobile phones. Further research should be conducted on students both from clinical and preclinical years to better assess the impact of the hospital environment on mobile phone contamination. Also, samples should be analyzed separately from different clinical wards as they can interfere with phone contamination. Reporting findings of other microorganisms on mobile phones will provide deeper insight into the extent of microbial burden. Comprehensive knowledge of the effectiveness of decontamination procedures is needed to develop adequate preventive strategies in a hospital environment.

## Supplementary Information

Supplementary Information.

## Data Availability

The datasets used and/or analyzed during the current study are available from the corresponding author upon reasonable request.

## References

[1] Cicciarella Modica D (2020). Taking screenshots of the invisible: A study on bacterial contamination of mobile phones from University students of healthcare professions in Rome, Italy. Microorganisms..

[2] Simmonds R, Lee D, Hayhurst E (2020). Mobile phones as fomites for potential pathogens in hospitals: Microbiome analysis reveals hidden contaminants. J. Hosp. Infect..

[3] Awwad AM (2021). Visual emotion-aware cloud localization user experience framework based on mobile location services. Int. J. Interact. Mobile Technol..

[4] Fard RH, Fard RH, Moradi M, Hashemipour MA (2018). Evaluation of the cell phone microbial contamination in dental and engineering schools: Effect of antibacterial spray. J. Epidemiol. Glob. Health..

[5] Olsen M (2020). Mobile phones represent a pathway for microbial transmission: A scoping review. Travel Med. Infect. Dis..

[6] Bodena D, Teklemariam Z, Balakrishnan S, Tesfa T (2019). Bacterial contamination of mobile phones of health professionals in Eastern Ethiopia: Antimicrobial susceptibility and associated factors. Trop. Med. Health..

[7] Pal S (2015). Mobile phones: Reservoirs for the transmission of nosocomial pathogens. Adv. Biomed. Res..

[8] Koydemir HC, Ozcan A (2017). Mobile phones create new opportunities for microbiology research and clinical applications. Future Microbiol..

[9] Meadow JF, Altrichter AE, Green JL (2014). Mobile phones carry the personal microbiome of their owners. PeerJ.

[10] Kurli R (2018). Cultivable microbial diversity associated with cellular phones. Front. Microbiol..

[11] Mushabati NA (2021). Bacterial contamination of mobile phones of healthcare workers at the University Teaching Hospital, Lusaka, Zambia. Infect Prev Pract..

[12] Galazzi A, Panigada M (2019). Microbiological colonization of healthcare workers' mobile phones in a tertiary-level Italian intensive care unit. Intensive Crit. Care Nurs..

[13] Hoog GS, Guarro J, Gene J, Figueras MJ (2000). Atlas of Clinical Fungi.

[14] Furuhata K (2016). Isolation, identification and antibacterial susceptibility of Staphylococcus spp. associated with the mobile phones of University students. Biocontrol Sci..

[15] Akinyemi KO, Atapu AD, Adetona OO, Coker AO (2009). The potential role of mobile phones in the spread of bacterial infections. J. Infect. Dev. Ctries..

[16] Sailo CV (2019). Pathogenic microbes contaminating mobile phones in a hospital environment in Northeast India: Incidence and antibiotic resistance. Trop. Med. Health..

[17] Banawas S (2018). Multidrug-resistant bacteria associated with cell phones of healthcare professionals in selected hospitals in Saudi Arabia. Can. J. Infect. Dis. Med. Microbiol..

[18] Pérez-Cano HJ, Reyes Santos MF, César Moreno BM (2019). Microbiota in mobile phones of medical ophthalmologists. Arch. Soc. Esp. Oftalmol. (Engl Ed)..

[19] Smibert OC (2018). Mobile phones, and computer keyboards: unlikely reservoirs of multidrug-resistant organisms in the tertiary intensive care unit. J. Hosp. Infect..

[20] Koscova J, Hurnikova Z, Pistl J (2018). Degree of bacterial contamination of mobile phone and computer keyboard surfaces and efficacy of disinfection with chlorhexidine digluconate and triclosan to its reduction. Int. J. Environ. Res. Public Health.

[21] Zakai S (2016). Bacterial contamination of cell phones of medical students at King Abdulaziz University, Jeddah, Saudi Arabia. J. Microsc. Ultrastruct..

[22] Lopez VA, Liew JY, Pahirulzaman KAP, Sim KY (2020). A study of microbial distribution and cell phone hygiene awareness at Universiti Malaysia Kelantan, Jeli Campus. Songklanakarin J. Sci. Technol..

[23] Cavari Y (2016). Healthcare workers mobile phone usage: A potential risk for viral contamination surveillance pilot study. Infect. Dis. (Lond).

[24] Ulger F, Dilek A, Esen S, Sunbul M, Leblebicioglu H (2015). Are healthcare workers' mobile phones a potential source of nosocomial infections? Review of the literature. J. Infect. Dev. Ctries..

[25] Heyba M (2015). A microbiological contamination of mobile phones of clinicians in intensive care units and neonatal care units in public hospitals in Kuwait. BMC Infect. Dis..

[26] Kordecka A, Krajewska-Kułak E, Łukaszuk C, Kraszyńska B, Kułak W (2016). Isolation frequency of Candida present on the surfaces of mobile phones and handsx. BMC Infect. Dis..

[27] Koroglu M (2015). Comparison of keypads and touch-screen mobile phones/devices as potential risk for microbial contamination. J. Infect. Dev. Ctries..

[28] Dubljanin E (2017). Epidemiology of onychomycosis in Serbia: A laboratory-based survey and risk factor identification. Mycoses.

[29] Smith N, Sehring M, Chambers J, Patel P (2017). Perspectives on non-neoformans cryptococcal opportunistic infections. J. Commun. Hosp. Intern. Med. Perspect..

[30] Jarros IC (2018). Yeasts from skin colonization are able to cross the acellular dermal matrix. Microb. Pathog..

[31] Cavalheiro M, Pais P, Galocha M, Teixeira MC (2018). Host-pathogen interactions mediated by MDR transporters in Fungi: As pleiotropic as it gets!. Genes (Basel)..

[32] Wiesmüller GA (2017). Abridged version of the AWMF guideline for the medical clinical diagnostics of indoor mould exposure. Allergo J. Int..

[33] Kotris I, Drenjančević D, Talapko J, Bukovski S (2017). Identification of microorganisms on mobile phones of intensive care unit health care workers and medical students in the tertiary hospital. Med. Glas (Zenica)..

[34] Olsen M (2021). Mobile phones of paediatric hospital staff are never cleaned and commonly used in toilets with implications for healthcare nosocomial diseases. Sci. Rep..

[35] Kuriyama A (2021). Prevalence of bacterial contamination of touchscreens and posterior surfaces of smartphones owned by healthcare workers: A cross-sectional study. BMC Infect. Dis..

