# Supplementary material for: Fungal contamination of medical students’ mobile phones from the University of Belgrade, Serbia: a cross-sectional study
Source: Sci Rep. 2022 Oct 7;12:16852. doi: 10.1038/s41598-022-21118-2 (PMC9540039; doi:10.1038/s41598-022-21118-2)
Supplement: Supplementary file 1 — Supplementary Information. [file 41598_2022_21118_MOESM1_ESM.docx]

Supplementary Table 1. Distribution of isolated fungi from students’ mobile phones by academic year of study.

| Isolated fungi | 4^th^ year  (n=158)  No. (%) | 2^nd^ year  (n=21)  No. (%) | Total  (n=179)  No. (%) | p-value* |
| --- | --- | --- | --- | --- |
| Yeasts | 65 (41.1) | 15 (71.4) | 80 (44.7) | 0.034 |
| Non-dermatophyte moulds | 91 (57.6) | 6 (28.6) | 97 (54.2) |  |
| Dermatophytes | 2 (1.3) | 0 (0.0) | 2 (1.1) |  |

*According to the chi-square test
